# Comparison of Platelet Ultrastructure and Elastic Properties in Thrombo-Embolic Ischemic Stroke and Smoking Using Atomic Force and Scanning Electron Microscopy

**DOI:** 10.1371/journal.pone.0069774

**Published:** 2013-07-16

**Authors:** Jeanette Noel Du Plooy, Antoinette Buys, Wiebren Duim, Etheresia Pretorius

**Affiliations:** 1 Department of Physiology, School of Medicine, Faculty of Health Sciences, University of Pretoria, Pretoria, South Africa; 2 Microscopy and Microanalysis, School of Medicine, Faculty of Health Sciences, University of Pretoria, Pretoria, South Africa; 3 Department of Neurology, School of Medicine, Faculty of Health Sciences, University of Pretoria, Pretoria, South Africa; University of Queensland, Australia

## Abstract

Thrombo-embolic ischemic stroke is a serious and debilitating disease, and it remains the second most common cause of death worldwide. Tobacco smoke exposure continues to be responsible for preventable deaths around the world, and is a major risk factor for stroke. Platelets play a fundamental role in clotting, and their pathophysiological functioning is present in smokers and stroke patients, resulting in a pro-thrombotic state. In the current manuscript, atomic force and scanning electron microscopy were used to compare the platelets of smokers, stroke patients and healthy individuals. Results showed that the elastic modulus of stroke platelets is decreased by up to 40%, whereas there is an elasticity decrease of up to 20% in smokers’ platelets. This indicates a biophysical alteration of the platelets. Ultrastructurally, both the stroke patients and smokers’ platelets are more activated than the healthy control group, with prominent cytoskeletal rearrangement involved; but to a more severe extent in the stroke group than in the smokers. Importantly, this is a confirmation of the extent of smoking as risk factor for stroke. We conclude by suggesting that the combined AFM and SEM analyses of platelets might give valuable information about the disease status of patients. Efficacy of treatment regimes on the integrity, cell shape, roughness and health status of platelets may be tracked, as this cell’s health status is crucial in the over-activated coagulation system of conditions like stroke.

## Introduction

Stroke is a serious and debilitating neurological disease, despite advances in treatments, it remains the second most common cause of death worldwide [1], [2]. Thrombo-embolic ischemic stroke is the most common form, associated with abnormal clotting. Similarly, tobacco smoke exposure continues to be responsible for preventable deaths around the world [3], and it is a major risk factor for stroke, predisposing the smoker to a pro-thrombotic, pro-inflammatory state. In both stroke and individuals who smoke, a hypercoagulable state is present, and platelets play a profound role during this state. Platelets become activated, change shape and undergo spreading and clump together to form a tight clot. These platelets are entrapped in a fibrin fiber mesh, together with red blood cells. Such clots may be difficult to break up during the normal fibrinolytic pathways, and this is the cause of thrombo-embolic events during stroke. In smoking, there is also a higher prevalence of thrombotic events. Because platelets form such a prominent part of hypercoagubility, their structure may give important information about the pathophysiology of thrombotic events. Particularly, their membrane integrity, elasticity and fluidity may be of importance.

Here, ultra-microscopy may provide additional information and one such method is atomic force microscopy (AFM), invented in 1986 [4] as well as scanning electron microscopy (SEM). AFM is an unique technique which enables the quantitative determination of micro-elastic properties of living cells in an aqueous environment [5] as well as in fixed and dehydrated cells [6]. Recent AFM techniques enables the solving of a number of problems in cell biomechanics, due to the simultaneous evaluation of local mechanical properties and topography of living cells at a very high spatial resolution and force sensitivity [7]. Imaging of elastic properties with the AFM is possible using force modulation [8], which has previously been applied to platelets [9], [10]. Quantitative analysis of elasticity can therefore be obtained by using force curves [11]. SEM analysis may provide important membrane structure information, particularly at high magnifications.

It is generally well accepted that cellular function is essentially determined by structure [7]. At various hierarchy levels found, the structural organization present in cells is characterized by mechanical properties. It is expected that cellular structure should vary, both in a variety of physiological processes (such as cell growth, differentiation, adhesion) and under pathogenesis (thrombosis, oxidative stress, inflammation) [7]. Three types of filamentous proteins are found in the cytoskeleton of cells; intermediate filaments, actin filaments (microfilaments), microtubules, and a wide variety of associated proteins. Together all these proteins are responsible for the general shape of the cell, the movement of the cell, and notably the generation of force. Additionally, they can have other functions for example the transport of vesicles [5].

Platelets shape change and activity after activation depends on the stimuli/agonists that are involved. The reorganization of the cytoskeleton in platelets is an important factor in the complex mechanisms found in thrombosis and haemostasis. The cytoskeleton is primarily responsible for regulating platelet shape [12]. The main cytoskeletal component in platelets is actin [13], [14] and the activation of platelets results in rapid changes in the amount of actin that is polymerized into actin filaments, as well as the organization of them. This process is essential for the beginning of platelet aggregation [14]. Various proteins are also involved in the reorganization of the cytoskeleton.

Clearly, the cytoskeleton plays a pivotal role in many aspects of the cell, including cell division, cell shape, cell adhesion and cell motility, therefore additional information about the stiffness of the cytoskeleton may prove useful in elucidating pathological conditions [5]. Platelet activation and spreading have previously been investigated using AFM [15]–[17]; however, the platelets found in pathological conditions like thrombo-embolic ischemic stroke and tobacco smokers have not yet been thoroughly studied. Therefore, the objective was to compare AFM images of healthy control subjects, smokers and stroke patients, as well as the quantitative determination of the local elastic properties of platelets in the groups. AFM results were compared and confirmed with SEM high magnification ultrastructural analysis.

## Materials and Methods

### Participants

Thrombo-embolic ischaemic stroke patients and “healthy” smokers were included in the current study. Exclusion criteria were acute illness, cancer, hepatic or renal dysfunction and the diagnosis were according to the WHO criteria and done by a Neurosurgeon according to the TOAST criteria. All patients underwent computed tomography (CT) scanning and standard thrombophilia screening. Ten smokers who have been smoking for at least 5 years and that were not taking any chronic medication, volunteered to take part in this study. Data concerning demographics and general health status were collected using a questionnaire and smoking was defined as smoking at least 5 cigarettes per day. Ten healthy individuals were used as controls. These participants were non-smokers, who did not use any chronic medication and did not have a history of thrombotic disease. 5 ml of blood was drawn into a citrate tube, from each participant. Ethical clearance was obtained for this study from the University of Pretoria Human Ethics Committee and informed consent was obtained from all participants. SEM micrographs from all participants were compared to our healthy individuals, stroke and smokers database, consisting of hundreds of platelet micrographs, and found to be a representative sample. All experiments were performed double-blind by 3 researchers.

### AFM Sample Preparation

Blood was collected in citrate tubes; a drop of the blood was placed on a glass cover slip, and spread out using circular motions for 30 second to allow activation and adhesion of the platelets to the glass cover slip. The coverslip was then washed with Dulbecco's Phosphate buffered saline (DPBS) buffer on a micro-plate shaker to separate and remove all other blood cells and plasma proteins from the glass cover slip and the platelets. Washed samples were then fixed in 2.5% glutaraldehyde DPBS with a pH of 7.4 for 15 minutes, followed by rinsing with DPBS and post-fixation with 1% osmium tetraoxide (OsO_4._) The samples were then again rinsed with DPBS and dehydrated with a series of ethanol. The procedures were completed by the dehydration of the material in hexamethyldisilazane (HMDS).

### AFM Imaging and Measurement

AFM imaging and quantitative determination of the local elastic properties of blood platelets was carried out using the Dimension Icon system manufactured by Bruker, operating in Peak Force™ QNM™ tapping mode. This mode operates by applying a controllable, constant force at each data point and using the resulting force-distant curve for the formation of a series of images; topography or height images are extracted from the cantilever deflection signal at the maximum contact force, modulus images are calculated from the slope of the retract curve near zero separation, adhesion images from the minimum of the retraction curve, deformation from the variation between the peak force point and the point where the force is zero and energy dissipation is calculated from the area between the approach and retract curve [18].

Additionally, and more importantly this mode is able to store the quantitative data of each force curve, enabling accurate modulus value acquirement at any preferred area. Silicon Nitride probes (ScanAsyst Air, Bruker, USA) with a force constant of 0.4 N/m (newton metre), a resonant frequency of 70 kHz (kilohertz) and a nominal tip radius of 9 nm was employed in all AFM measurements.

Cells of each sample were scanned at the following fields; 10 µm by 10 µm (this is similar to a ±25000–30000 times magnification) and 1 µm by 1 µm. A field of 0.25 µm by 0.25 µm was also scanned on the central (highest) region of the cell where three random scan lines of force-distance curves was recorded, an average of 1000 force curves was taken per cell.

Only force curves with a goodness of fit of 0.85 and above was used for elasticity measurements. The statistical significance of the difference between calculations was determined using one-way analysis of variance. A 2-tailed P value of less than 0.05 was considered to be significant. The elastic Young’s modulus of the cells was calculated from the force-distance curves using the Derjaguin–Muller–Toporov (DMT) theory [19], which is based on the well-known Hertz models geometry, but considers that additional attractive interactions occur [18].

### Scanning Electron Microscopy (SEM)

Platelet rich plasma (PRP) smears were prepared on glass cover slips for SEM analysis. All samples were viewed with a ZEISS FEG scanning electron microscope at 1 kV. SEM micrographs from the current study were compared to our database of hundreds of smokers, stroke and healthy individual platelets and found to be comparable.

## Results

The Young’s modulus for the fixed and dehydrated control platelets were in the range of 30–130 MPa (megapascal), which is comparable to results found in similar studies [20] and approximately a thousand times higher than living “wet” platelets, 1–50 kPa (kilopascal) [5].

The elastic modulus of platelets form stroke patients, is decreased by about 40%, whereas the modulus form smokers platelets, is decreased by approximately 20% (Table 1). This signifies a biophysical alteration of the platelets, resulting in “softer” platelets, which is indicative of cytoskeletal rearrangement.

**Table 1:** Average values of Young’s modulus (E, MPa).

	Mean	Standard Deviation	Median	Standard Error
**Control**	57.56	27.63	53.6	0.23
**Stroke**	34.91	20.40	31.6	0.23
**Smoker**	44.65	14.43	43.8	0.16

**Figure 1:**
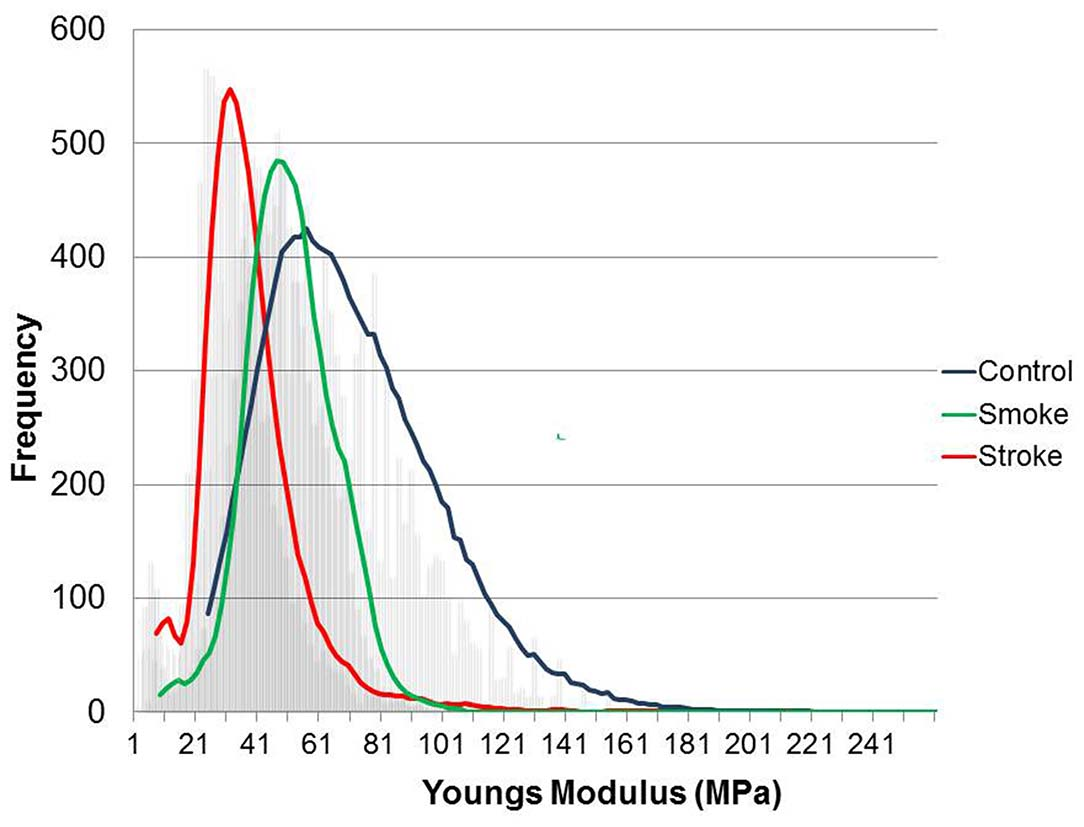
Distribution of Young’s moduli obtained from control platelets (blue), stroke platelets (red) and cigarette smoke platelets (green). Stroke platelets are approximately 40% less elastic and smokers’ platelets are approximately 20% less elastic than healthy platelets.

**Figure 2:**
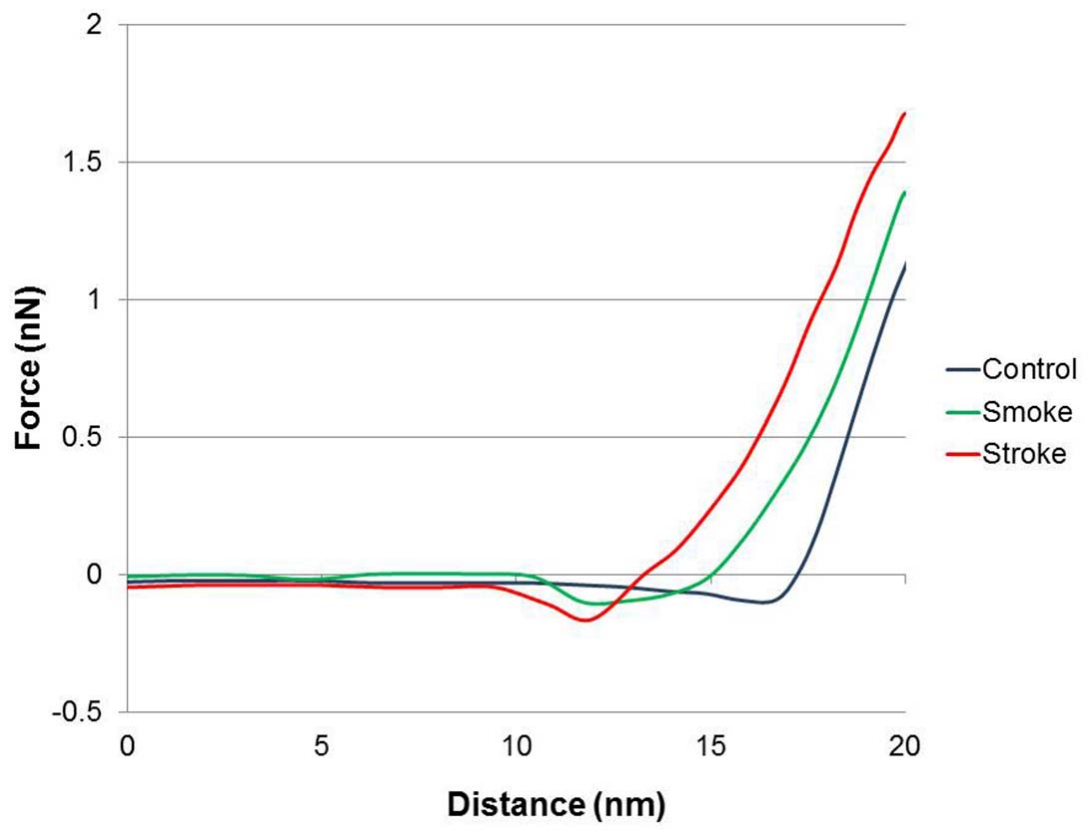
Force-Distance curves obtained on control platelets (blue), stroke platelets (red) and cigarette smoke platelets (green). Force-Distance curves show the AFM cantilelver deflection range on the platelet surface. The deflection is the most in healthy platelets.

Figure 1 illustrates these findings, by showing a graph of the frequency distribution of Young’s moduli obtained from the different groups. All the groups presented with a relatively normal distribution shape, is roughly centred on the average modulus values, and also seen in Table 1. However, interestingly with increasing softness (control>cigarette smoke>stroke) the range of moduli decreases, which suggest that the total platelet population is affected. Figure 2 gives representative examples of force distance curves, recorded using control platelets (blue), stroke platelets (red) and cigarette smoke platelets (green). The curves consist of two main regions; the flat region, where the tip is not yet touching the specimen and the sloped region where the tip comes into contact with, and therefore applies a force to the cell. The tip will deform the cells to different extents, depending on the stiffness of the sample; softer samples will deform more easily, therefore presenting with a less linear shape, and a smaller slope, due to the increased cell compliance. As can be seen in Figure 2 the healthy platelet present with a linear shaped curve and a sharp incline, whereas the curve of the stroke and cigarette smoke platelets becomes increasingly non-linear, with less of an gradient.

**Figure 3:**
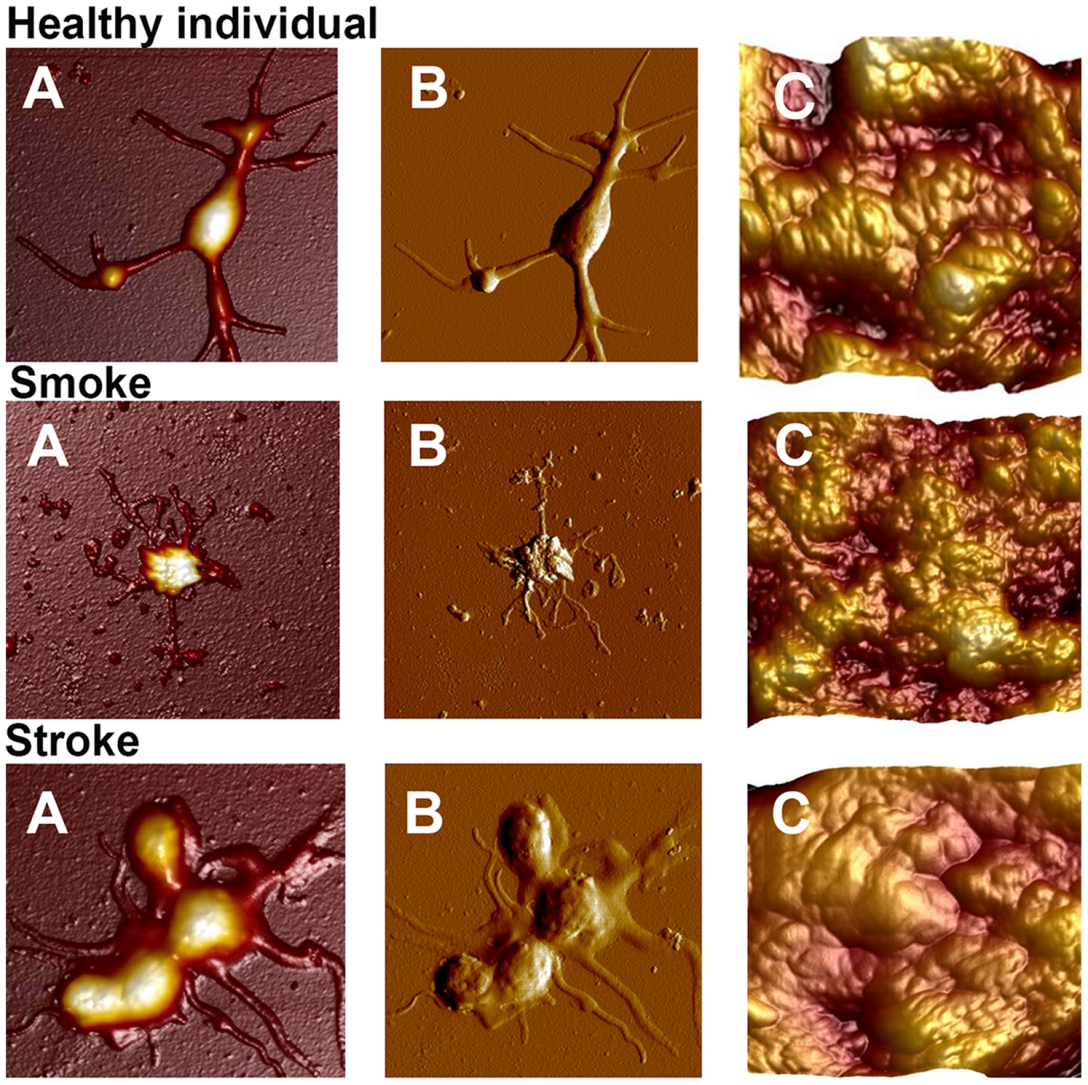
AFM images of a platelets from a healthy individual, stroke patient and smoker. **A)** Height image, x–y scale: 10 µm by 10 µm, z scale: 1 µm, **B)** Error image, **C)** Height image of the platelet membrane, x–y scale: 1 µm by 1 µm, z scale: 0.2 µm.

Figure 3 represents the AFM images obtained in this study. When looking at figures A and B, it appears as if the stroke patient’s platelet is activated, and also swollen, when compared to healthy platelets. Platelets from cigarette smokers appear to be activated, however, here the platelets seem shrunken, and the membrane appears irregular. Correlating to the lower magnification images the high magnification images of the platelet membrane (C) the stroke patients platelet appears more “stretched” where the smokers appears to be more “shrivelled” when compared to the control.

## Discussion

From the quantitative data, conclusions regarding activation can be made. The elastic modulus (Young’s modulus Table 1) in stroke patients is decreased by 40%, whereas elastic modulus in smokers is decreased by 20%. This indicates a biophysical alteration of the platelets making them softer. This softer appearance is indicative of cytoskeletal rearrangement. Platelet activation is partly initiated and continued via various forms of cytoskeletal rearrangement. It is therefore expected that both stroke patients and smokers’ platelets would be in an altered state. Here the modulus of stroke patients is decreased more than that of smokers. This is an indication that the platelets of stroke patients are in a more advanced activated state than the smokers group. Furthermore, modulus of the smokers group is decreased with 20% compared to healthy individuals, and is indicative of irregular platelet morphology and impaired function in the smokers group. As expected, progressive worsening of platelet membrane structure is therefore noted, with stroke platelets having the weakest pathology. The force curves show the cantilever deflection when a force is applied to the platelet. The deflection is more in healthy cells. Therefore, Figure 2 shows that both the stroke and smoker platelets have less deflection than healthy platelets, making them softer. This confirms results from the Young’s modulus (Figure 1).

Both SEM and transmission electron microscopy (TEM) as well as video-enhanced light microscopy has proven useful to study the organization of activated platelets, a strong correlation has been found in the topography and the underlying cytoskeleton [21]–[24]. According to Radmacher and co-workers, AFM figures obtained of platelets can show the underlying organization of cellular features. The authors made use of the following nomenclature: pseudo-nucleus, inner filamentous zone, outer filamentous zone, and the cortex [5]. The pseudo-nucleus consists primarily of small vesicles filled with proteins, granules and cytosol. This area is known to be the softest part of the cell. The inner filamentous zone contains the contractile apparatus of the platelet. Stiffer parts found may correspond to the areas with a dense network of filamentous actin and myosin adjacent to softer regions where a lower concentration of the polymers is present. The outer filamentous zone consists primarily of microtubules and bundles of actin filaments. A dense homogeneous network consisting of short actin filaments is found in the cortex [5].

Deduced from the above-mentioned literature, and the current results, where there is a changed platelet modulus found in smokers and stroke patients, it seems as if the softer appearance of the platelets is likely due to the release or secretion of granules (alpha-granules, dense granules) by the activated platelets. Radmacher and co-workers described the pseudo-nucleus as the softest part of the platelet; therefore the presence of granules, vesicles and cytosol is responsible for this [5]. Platelet secretion or exocytosis releases molecules at the site of injury to activate other cells or to facilitate cellular adhesion. Platelets secrete molecules from intracellular granules. These molecules play central roles in haemostasis, thrombosis, and vascular remodelling [25], [26]. Therefore, the softer appearance of the platelets of stroke patients and smokers may be indicative of an increased production and release of granules, as well as a breakdown in the organization of the cytoskeleton/contractile apparatus.

Figure 3 shows images obtained from the different groups using AFM. The images show platelets from a healthy individual, stroke patient and smoker. Figure 3A) shows the height images, Figure 3B) shows the error image and Figure 3C) shows height image of the platelet membranes. The peak force error image is generated by signals of the difference in values between the peak force set-point and the desired value of the set-point, which is attained from the closed-loop feedback system. This drives the z-piezo to adjust the height and force – thereby realizing the desired set-point. An image similar to low magnification ultra-microscopy is therefore obtained. When looking at platelets from healthy individuals, they have some pseudopodia formation (panel A and B). This might be due to the preparation process and attachment to the glass cover slip. Their membranes also appear globular (panel C).

Platelets from the smokers group appear to be activated and spreading around the main body of the platelet is seen as granular deposits (panel A and B). However, in contrast to the stroke platelets (A and B), it seems as if contraction of the platelet itself occurs; with the globular structure of the membrane appearing irregular and compromised (panel C).

Platelets of the stroke patients, it appears as if they are more activated, with prominent spreading around the body of the platelet. These platelets are also more swollen, when compared to that of healthy platelets and the shrunken platelets from smokers. This swelling may indicate the presence of necrosis. Pretorius and co-workers in 2012 suggested that necrotic platelets are present in stroke patients [27]. Extensive cell spreading is also clearly visible and not present in the platelets from healthy individuals or smokers.

Smokers platelets may have a changed fluidity – this was previously mentioned by Padmavathi and co-workers found in 2010 that smoking induces alterations in platelet membrane fluidity and NA^+^/K^+^-ATPase activity, this was confirmed ultrastructurally by Pretorius and co-workers in 2012 [28], [29].

**Figure 4:**
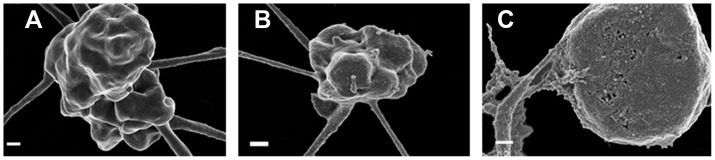
Scanning electron microscopy micrographs of A) healthy individual; B) smoker; C) stroke patient. Scale = 200 nm.

**Figure 5:**
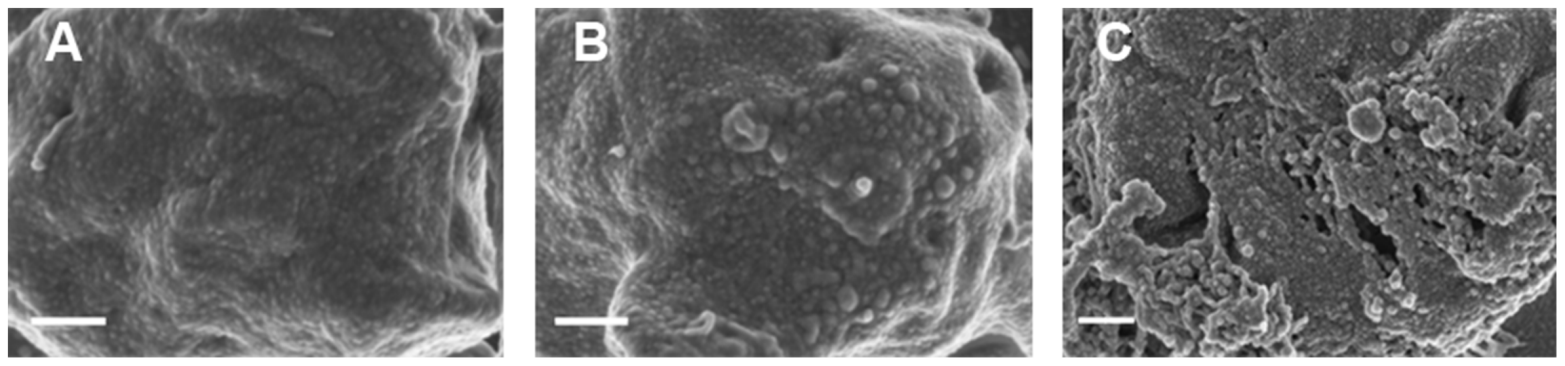
High magnification scanning electron microscopy micrographs of membranes A) healthy individual; B) smoker; C) stroke patient. Scale = 200 nm.

SEM micrographs also confirm the changed platelet structure (Figure 4). Platelets of smokers appear slightly smaller but still with a bulbous structure as seen in healthy platelets (Figure 4A and B). Stroke platelets are all clearly swollen and membrane breaks can be seen (Figure 4C). The high SEM magnifications of the platelet membranes also show prominent differences (Figure 5). Healthy platelets have a smooth membrane structure with slight bulbous areas curving over the platelet body (Figure 5A). The platelet membranes from smokers change to form larger globular areas (Figure 5B); while the platelet membranes in stroke show membrane breakages and appear much more granular when compared to the platelet membranes of the healthy individuals. We therefore suggest that a slight cytoskeletal re-arrangement in seen in platelets of smokers, while in stroke platelets this cytoskeletal re-arrangement seems irreversible. The micrographs shown here are representative of most of the platelets in the smokers and stroke individuals. These SEM high magnification results therefore support and confirm the AFM results (Figure 3), particularly when studying the high membrane magnifications (Figure 4 and 5).

Both the stroke and smokers’ platelets are more activated than the healthy individual group, with prominent cytoskeletal rearrangement involved - to a more severe extent in the stroke than in the smokers. Importantly, this is a confirmation of the extent of smoking as risk factor for stroke. This is confirmed using both AFM and SEM analysis. We conclude by suggesting that the combined AFM and SEM analyses of platelets might give valuable information about the disease status of patients. The ultrastructural changes discussed here are not visible using a traditional light microscope. Efficacy of treatment regimes on the integrity, cell shape, roughness and health status of platelets may be tracked, as this cell’s health status is crucial in the over-activated coagulation system of conditions like stroke.

### Ethical Approval Disclosure

Ethical approval was granted at the University of Pretoria (HUMAN ETHICS COMMITTEE: FACULTY OF HEALTH SCIENCES). All human blood samples obtained were analysed at the University of Pretoria and all participants filled in informed consent forms.
